# Choice of library preparation affects sequence quality, genome assembly, and precise *in silico* prediction of virulence genes in shiga toxin-producing *Escherichia coli*

**DOI:** 10.1371/journal.pone.0242294

**Published:** 2021-03-24

**Authors:** Julie Haendiges, Karen Jinneman, Narjol Gonzalez-Escalona

**Affiliations:** 1 Center for Food Safety and Applied Nutrition, Food and Drug Administration, College Park, Maryland, United States of America; 2 Office of Regulatory Affairs, Food and Drug Administration, Bothell, Washington, United States of America; University of Texas at San Antonio, UNITED STATES

## Abstract

Whole genome sequencing (WGS) provides essential public health information and is used worldwide for pathogen surveillance, epidemiology, and source tracking. Foodborne pathogens are often sequenced using rapid library preparation chemistries based on transposon technology; however, this method may miss random segments of genomes that can be important for accurate downstream analyses. As new technologies become available, it may become possible to achieve better overall coverage. Here we compare the sequence quality obtained using libraries prepared from the Nextera XT and Nextera DNA Prep (Illumina, San Diego, CA) chemistries for 31 Shiga toxin-producing *Escherichia coli* (STEC) O121:H19 strains, which had been isolated from flour during a 2016 outbreak. The Nextera DNA Prep gave superior performance metrics including sequence quality, assembly quality, uniformity of genome coverage, and virulence gene identification, among other metrics. Comprehensive detection of virulence genes is essential for making educated assessments of STECs virulence potential. The phylogenetic SNP analysis did not show any differences in the variants detected by either library preparation method which allows isolates prepared from either library method to be analysed together. Our comprehensive comparison of these chemistries should assist researchers wishing to improve their sequencing workflow for STECs and other genomic risk assessments.

## Introduction

Whole genome sequencing (WGS) has been used in public health surveillance and outbreak detection of foodborne illnesses since 2012 [1], enabling researchers to perform phylogenetic analyses of strains, determine serotypes, identify virulence factors, and document antimicrobial resistance. Many of these WGS analyses were performed using enzymatic fragmentation by transposon enzymes [2]. While this method of DNA library preparation has been extremely useful for determining phylogenies and source tracking, it can be biased in certain regions of the genomes, potentially overlooking randomly distributed segments of sequence that might be important for downstream analyses [3–5]. This bias could be especially problematic when analysing organisms such as Shiga toxin-producing *Escherichia coli* (STEC), whose complex genomes contain multiple repetitive elements (i.e. insertion sequences, phages and plasmids) [5–7].

Classifying pathogenic *E*. *coli* depends on detecting the presence of pathogenicity islands, virulence genes, and plasmids that cause diarrheal illness. In order for a STEC to cause human illness, the strain must carry genes that allow for attachment, colonization, and production of Shiga toxin [8–11]. Although most of those virulence genes are found in the chromosome, others are typically carried on the virulence plasmid [8, 10, 12]. Most STECs contain such virulence plasmids, that may differ in gene content, and the assortment of virulence genes can affect clinical outcomes and detecting these are highly important for surveillance [13, 14]. Thus, as newer or alternative methods of DNA library preparation become available, it is important to determine whether these can provide better breadth of coverage. This highlights the importance of producing a library that contains the complete STEC genome in order to make an informed decision about the public health impact of the analyzed strain.

Here we explore whether an updated DNA library preparation chemistry (Nextera DNA Prep) that uses magnetic-bead linked transposomes (BLT) can more rapidly and accurately capture the genomic data from most genomic regions in a set of STECs (Thirty-one O121:H19 strains, which had been isolated from flour during a 2016 outbreak), than an earlier DNA library preparation method (Nextera XT) [15]. These two DNA library preparation kits are enzymatic fragmentation chemistries but there are three main differences between these kits: 1) the fragmentation process of the transposome, 2) dual size selection step, and 3) the quality of the indices [2]. Our evaluation of these kits will be based on the following metrics: sequencing metrics (Q30, insert size, and data output per sequencing run), assembly quality (N50, breath of coverage, and total genome size), *in silico* determination of serotype, MLST, AMR genes, and virulence genes, as well as single nucleotide polymorphism (SNP) phylogenetic analysis.

## Materials and methods

### Bacterial strains and DNA preparation

The strains were isolated from various types of flour by the U.S. Food and Drug Administration ORA Pacific Regional laboratory as part of a Federal public health multistate *Escherichia coli* investigation. The isolates were grown overnight in trypticase soy broth (TSB) (Becton, Dickinson and Company, Franklin Lakes, NJ) at 37°C and genomic DNA was extracted using the Qiagen DNeasy Blood and Tissue Kit (Qiagen, Valencia, CA). The concentration of resultant DNA was determined using a Qubit double-stranded DNA BR assay kit and a Qubit fluorometer (ThermoFisher Scientific, Waltham, MA), according to the manufacturer’s instructions, then stored at -20°C until use. The 31 shiga toxin-producing strains used in this study are listed in Table 1.

**Table 1:** Metadata for the 31 STEC strains used in this study.

CFSAN No.	Isolate name	Source	Location	Date of Collection	Serotype	Biosample Accession	ST^a^
CFSAN051458	FNW19M81	All-Purpose Flour	USA:MO	04/2016	O121:H19	SAMN05215988	655
CFSAN051559	FNW19M89	All-Purpose Flour	USA:AZ	06/2016	O121:H19	SAMN05245391	655
CFSAN051560	FNW19M90	All-Purpose Flour	USA:AZ	06/2016	O121:H19	SAMN05245392	655
CFSAN051561	FNW19M91	All-Purpose Flour	USA:AZ	06/2016	O121:H19	SAMN05245393	655
CFSAN051562	FNW19M92	All-Purpose Flour	USA:AZ	06/2016	O121:H19	SAMN05245394	655
CFSAN051563	FNW19M93	All-Purpose Flour	USA:AZ	06/2016	O121:H19	SAMN05245395	655
CFSAN051564	FNW19M94	All-Purpose Flour	USA:AZ	06/2016	O121:H19	SAMN05245396	655
CFSAN051565	FNW19M95	All-Purpose Flour	USA:AZ	06/2016	O121:H19	SAMN05245618	655
CFSAN051566	FNW19M96	All-Purpose Flour	USA:AZ	06/2016	O121:H19	SAMN05245621	655
CFSAN051567	FNW19M97	All-Purpose Flour	USA:AZ	06/2016	O121:H19	SAMN05245624	655
CFSAN051568	FNW19M98	All-Purpose Flour	USA:AZ	06/2016	O121:H19	SAMN05245744	655
CFSAN051758	FNW19N01	Unbleached White Flour	USA:MI	06/2016	O121:H19	SAMN05289759	655
CFSAN051759	FNW19N02	Unbleached White Flour	USA:MI	06/2016	O121:H19	SAMN05289761	655
CFSAN051760	FNW19N03	Unbleached White Flour	USA:MI	06/2016	O121:H19	SAMN05289763	655
CFSAN051761	FNW19N04	Unbleached White Flour	USA:MI	06/2016	O121:H19	SAMN05289764	655
CFSAN051762	FNW19N05	Unbleached White Flour	USA:MI	06/2016	O121:H19	SAMN05289767	655
CFSAN051763	FNW19N06	Unbleached White Flour	USA:MI	06/2016	O121:H19	SAMN05289769	655
CFSAN051764	FNW19N07	Unbleached White Flour	USA:MI	06/2016	O121:H19	SAMN05289771	655
CFSAN051765	FNW19N08	Unbleached White Flour	USA:MI	06/2016	O121:H19	SAMN05289773	655
CFSAN051766	FNW19N09	Unbleached White Flour	USA:MI	06/2016	O121:H19	SAMN05289774	655
CFSAN051767	FNW19N10	Unbleached White Flour	USA:MI	06/2016	O121:H19	SAMN05289776	655
CFSAN051768	FNW19N11	Unbleached White Flour	USA:MI	06/2016	O121:H19	SAMN05289777	655
CFSAN051769	FNW19N12	Unbleached White Flour	USA:MI	06/2016	O121:H19	SAMN05289778	655
CFSAN051770	FNW19N13	Unbleached White Flour	USA:MI	06/2016	O121:H19	SAMN05289779	655
CFSAN051771	FNW19N14	Unbleached White Flour	USA:MI	06/2016	O121:H19	SAMN05289780	655
CFSAN051772	FNW19N15	Unbleached White Flour	USA:MI	06/2016	O121:H19	SAMN05289781	655
CFSAN052204	FNW19N17	Enriched White Flour	USA:OK	06/2016	O121:H19	SAMN05294031	655
CFSAN052205	FNW19N18	Enriched White Flour	USA:OK	06/2016	O121:H19	SAMN05294032	655
CFSAN052206	FNW19N19	Enriched White Flour	USA:OK	06/2016	O121:H19	SAMN05294033	655
CFSAN052207	FNW19N20	Enriched White Flour	USA:OK	06/2016	O121:H19	SAMN05294034	655
CFSAN052208	FNW19N21	Enriched White Flour	USA:OK	06/2016	O121:H19	SAMN05294035	655

^a^ http://enterobase.warwick.ac.uk/species/index/ecoli.

### Closure of reference genome using MinION and Flye

For further bioinformatic analyses a closed reference strain was necessary. Strain FNW19M81 was obtained and grown overnight in TSB at 37°C. The genomic DNA was isolated using the Maxwell RSC Cultured Cells DNA kit (Promega, Madison, WI) following the manufacturer’s protocols with the addition of RNase A (ThermoFisher Scientific, Waltham, MA) treatment. Closure of this genome was performed using both short and long read sequencing technology. The short-read sequencing library was prepared using Illumina DNA Prep and the long-read sequencing library was prepared using the Ligation Sequencing Kit (SQK-LSK108) (Oxford Nanopore Technologies, Oxford, UK). Short read sequencing was performed using Illumina v3 sequencing reagents on an Illumina MiSeq while the long reads were sequenced on a FLO-MIN106 (R9.4) flowcell for 48 hours on an Oxford Nanopore MinION device. The long-reads were live basecalled using Albacore v2.0.1 included in the MinKNOW software (v1.10.11). All reads below 5,000 basepairs in length were removed from further analysis. The assembly procedure was performed as described in [16] with the following alterations: the assembly of the long reads was performed with Flye v1.6 [17] instead of Canu [18] and for the hybrid assembly Unicycler v0.4.8 [19] was used instead of SPAdes [20]. The genome was deposited in GenBank under accession number CP051631 and CP051632. The final assembly of the chromosome and plasmid were annotated using Prokka v1.13 [21].

### Library preparation and whole genome sequencing

In order to compare the data generated by the two library preparation kits, the same DNA extract was used as input for both library preparations. The DNA library preparations were performed manually with the recommended concentrations of DNA for each protocol was used (1 ng for XT and 1–500 ng for Prep). The DNA input value for each strain can be found in Table 2. An Illumina MiSeq benchtop sequencer (Illumina, San Diego, CA) was used to sequence the two library preparations with v3 sequencing chemistry with 2x250 bp pair-end reads.

**Table 2:** Assembly statistics by DNA library kit for each strain.

Strain	Library Method	SRA Accession	Total DNA Input (ng)	Sequencing Depth (X)	> Q30 Avg. Read lengths	Average insert size	# Contigs >500 bp	N50 (bp)	Total Genome Size (bp)
CFSAN051458	XT	SRR3657284	1	64	134	251	203	122,571	5,163,585
	PREP	SRR11508024	12	108	230	314	234	137,900	5,254,858
CFSAN051559	XT	SRR3747656	1	108	160	260	233	124,862	5,178,429
	PREP	SRR11507285	13	98	226	304	230	134,860	5,185,305
CFSAN051560	XT	SRR3747657	1	70	162	265	279	90,323	5,240,574
	PREP	SRR11508023	12	112	231	323	225	149,318	5,252,726
CFSAN051561	XT	SRR3747658	1	37	166	243	250	122,675	5,167,280
	PREP	SRR11507295	12	111	231	328	223	149,318	5,191,727
CFSAN051562	XT	SRR3747659	1	32	166	262	250	122,457	5,169,307
	PREP	SRR11507316	83	92	238	359	213	161,492	5,185,472
CFSAN051563	XT	SRR3747660	1	121	164	251	267	114,952	5,234,755
	PREP	SRR11507718	17	97	233	326	226	136,153	5,246,434
CFSAN051564	XT	SRR3747661	1	59	169	265	231	122,671	5,173,045
	PREP	SRR11507313	93	118	236	327	203	161,492	5,182,363
CFSAN051565	XT	SRR3747662	1	32	159	266	229	118,199	5,168,254
	PREP	SRR11507300	81	101	237	339	247	136,153	5,201,361
CFSAN051566	XT	SRR3747677	1	81	166	271	232	134,860	5,242,198
	PREP	SRR11507286	14	124	232	333	225	136,153	5,252,920
CFSAN051567	XT	SRR3747663	1	173	168	245	222	134,860	5,176,821
	PREP	SRR11507293	13	102	233	324	212	136,153	5,179,935
CFSAN051568	XT	SRR3747664	1	74	158	253	232	92,524	5,174,090
	PREP	SRR11506703	124	101	239	343	192	161,492	5,180,491
CFSAN051758	XT	SRR3713425	1	107	189	251	245	134,860	5,233,452
	PREP	SRR11507309	11	131	228	320	230	136,153	5,255,567
CFSAN051759	XT	SRR3713426	1	109	195	268	218	124,722	5,174,121
	PREP	SRR11507292	12	99	230	326	214	136,153	5,185,045
CFSAN051760	XT	SRR3713427	1	123	166	257	218	136,153	5,174,288
	PREP	SRR11507315	11	98	227	323	212	149,318	5,180,971
CFSAN051761	XT	SRR3713429	1	113	192	236	272	118,379	5,230,338
	PREP	SRR11507717	122	104	239	347	211	134,860	5,241,487
CFSAN051762	XT	SRR3713430	1	115	168	250	261	111,929	5,232,272
	PREP	SRR11647983	27	121	226	322	205	136,153	5,243,799
CFSAN051763	XT	SRR3713431	1	72	230	331	260	100,914	5,220,779
	PREP	SRR11508014	101	109	239	343	214	136,153	5,242,282
CFSAN051764	XT	SRR3713432	1	117	220	295	212	147,488	5,180,308
	PREP	SRR11647979	12	86	223	310	210	136,153	5,182,293
CFSAN051765	XT	SRR3713433	1	90	229	354	225	147,488	5,242,987
	PREP	SRR11508027	81	98	237	341	236	161,492	5,249,988
CFSAN051766	XT	SRR3713434	1	112	183	270	224	122,671	5,239,845
	PREP	SRR11507380	112	113	239	343	210	136,153	5,244,820
CFSAN051767	XT	SRR3713435	1	107	169	242	222	134,860	5,174,859
	PREP	SRR11508026	105	121	240	362	203	149,318	5,183,709
CFSAN051768	XT	SRR3713436	1	90	205	281	221	134,860	5,180,416
	PREP	SRR11507722	78	98	239	348	213	136,153	5,180,511
CFSAN051769	XT	SRR3713563	1	108	197	266	244	132,264	5,244,997
	PREP	SRR11507308	82	94	240	357	219	136,153	5,246,062
CFSAN051770	XT	SRR3713437	1	110	182	261	224	134,860	5,178,190
	PREP	SRR11506716	69	90	239	360	215	149,318	5,184,468
CFSAN051771	XT	SRR3713438	1	111	221	312	241	147,488	5,194,013
	PREP	SRR11507290	30	103	236	328	243	136,153	5,203,407
CFSAN051772	XT	SRR3713439	1	105	198	269	232	134,860	5,239,307
	PREP	SRR11507381	66	94	239	348	223	161,492	5,252,618
CFSAN052204	XT	SRR3743154	1	101	180	259	238	134,860	5,242,017
	PREP	SRR11507299	70	97	239	361	214	136,153	5,241,473
CFSAN052205	XT	SRR3743156	1	89	177	266	231	134,860	5,241,355
	PREP	SRR11507282	59	94	238	361	217	136,153	5,243,446
CFSAN052206	XT	SRR3743157	1	165	192	271	231	134,860	5,246,208
	PREP	SRR11507724	72	86	240	370	213	161,492	5,243,257
CFSAN052207	XT	SRR3743158	1	116	203	266	243	134,860	5,243,419
	PREP	SRR11647986	88	96	240	357	207	161,492	5,243,634
CFSAN052208	XT	SRR3743159	1	111	193	266	225	134,860	5,180,663
	PREP	SRR11647987	149	111	240	352	209	136,153	5,178,696

(XT = Nextera XT; PREP = Nextera DNA Prep)

### Bioinformatic analysis

Two *de novo* assemblies were generated from the reads of each DNA library using SPAdes v3.13.0 [20], using k-mer lengths of [21, 33, 55, 77, 99, 127], and the options “—careful” and “—only-assembler”. All assemblies were quality checked using Quast v5.0 [22] and evaluated for the number of contigs, largest contig length, and N50. All contigs with a length below 500 bp were trimmed from the final assemblies before proceeding to analysis with AMRfinder.

The initial analysis and identification of the strains were performed using an *in silico E*. *coli* MLST approach, based on the information available at the *E*. *coli* MLST website (http://mlst.warwick.ac.uk/mlst/dbs/Ecoli) and using Ridom SeqSphere+ software v2.4.0 (Ridom; Münster, Germany) (http://www.ridom.com/seqsphere). Seven housekeeping genes (*adk*, *fumC*, *gyrB*, *icd*, *mdh*, *recA*, and *purA*), described previously for *E*. *coli* [23], were used for MLST analysis. The same *E*. *coli* MLST database was also used to assign numbers for alleles and sequence types (STs). The serotype of each strain analyzed in this study was confirmed using the genes deposited in the Center for Genomic Epidemiology (http://www.genomicepidemiology.org) for *E*. *coli* as part of their web-based serotyping tool (SerotypeFinder 1.1 https://cge.cbs.dtu.dk/services/SerotypeFinder) [24]. Each whole genome sequence was screened for O-type or H-type genes.

Virulence genes, stress genes, and antimicrobial resistance (AMR) genes were identified using the AMRFinder v3.6.10 command line tool [25]. All assemblies were analyzed using the “—plus” option to include *E*. *coli* virulence genes and stress tolerance genes. This database contains over 600 virulence reference sequences, 200 stress reference sequences, and 6,000 AMR reference sequences. The virulence genes included in the database represent a repertoire of genes found in different *E*. *coli* pathotypes (ETEC, STEC, EAEC, and EPEC) in order to detect any known possible *E*. *coli* hybrid present [26].

### Phylogenetic analysis

The phylogenetic relationship of the strains was assessed by the CFSAN Single Nucleotide Polymorphism (SNP) Pipeline v2.2.1 [27]. The closed genome of FNW19M81 was used as the reference for analysis. To serve as the outgroups in our analysis, we used three clinical strains not associated with the 2016 outbreak. To evaluate if SNP calls were affected by library preparation, the raw reads from both library preparations, for all strains, was analyzed. A maximum likelihood tree was generated using RAxML v8.2.9 [28] from the SNP matrix, using 500 bootstraps and the GTRCAT substitution model to identify the best tree.

### Nucleotide sequence accession numbers

The draft genome sequences for all 31 STEC strains used in our analyses are available in GenBank under the accession numbers listed in Table 2.

## Results

### Reference strain analysis

In order to accurately assess performance differences between the two DNA library preparations, we closed one representative O121:H19 outbreak strain. The complete closed circular genome for strain FNW19M81 contained one chromosome (CP051631) of length 5,391,339 bp (50.7% GC) and a single plasmid (CP051632) of length 81,965 bp (46.3% GC). This strain carried 19 out of the 94 virulence genes tested by *in silico* analysis, three of which: enterohaemolysin (*ehxA–*QJE08818.1), serine protease autotransporter (*espP*–QJE08794.1), and Toxin B (*toxB*–QJE08790.1) were located on the plasmid. We used this strain to test for inclusivity of all virulence, AMR, and stress tolerance genes tested.

### Evaluation of assembly quality

**Fig 1:**
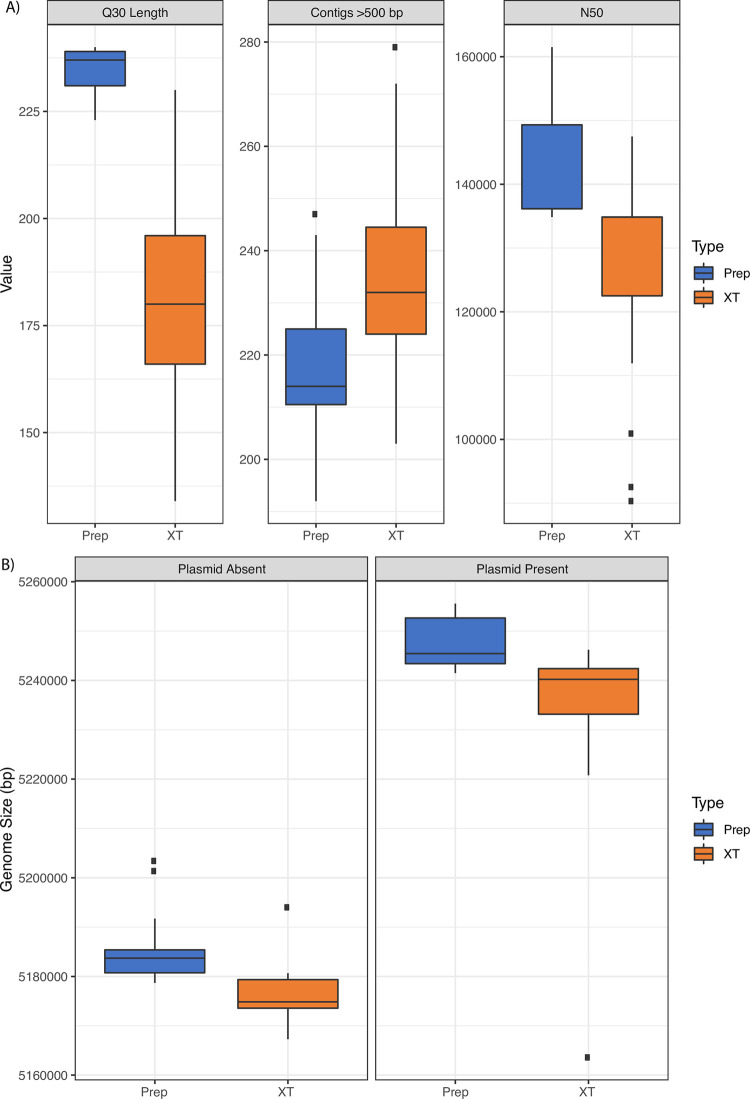
Comparison of the statistics for DNA library kit. The DNA Prep kit resulted in sequencing reactions that rendered the better read length quality, assemblies and N50s. A) Q30 read length, Contigs, and N50 for the data prepared with the two library preparation methods. B) The overall genome size distribution of isolates with and without the plasmid based on library preparation.

We used the same DNA extract for both library methods, to ensure direct comparison of the results and quality of the different library preparations. Thus, two *de novo* assemblies were produced for each of the thirty one strains, one for each library preparation (XT and DNA Prep). The quality of these assemblies was evaluated by examining the Quast and SPAdes outputs, focusing mainly on the values of contigs above >500 bp, total genome size, N50, average insert size, and Q30 read length quality. These results are shown in Table 2. The Q30 read length distribution for DNA Prep libraries were more consistent and larger in comparison to XT as seen in Fig 1A. Additionally, these libraries assembled into less contigs with a lower median contig number and had a higher N50 (Fig 1A). The assemblies from XT ranged from 203 to 279 contigs while the assemblies from DNA Prep ranged from 192 to 249 total contigs. The Prep assemblies had larger genome size distribution than the XT assemblies in both those isolates with and without the plasmid (Fig 1B) with the median genome size and the lower quartile larger for the Prep assemblies.

**Fig 2:**
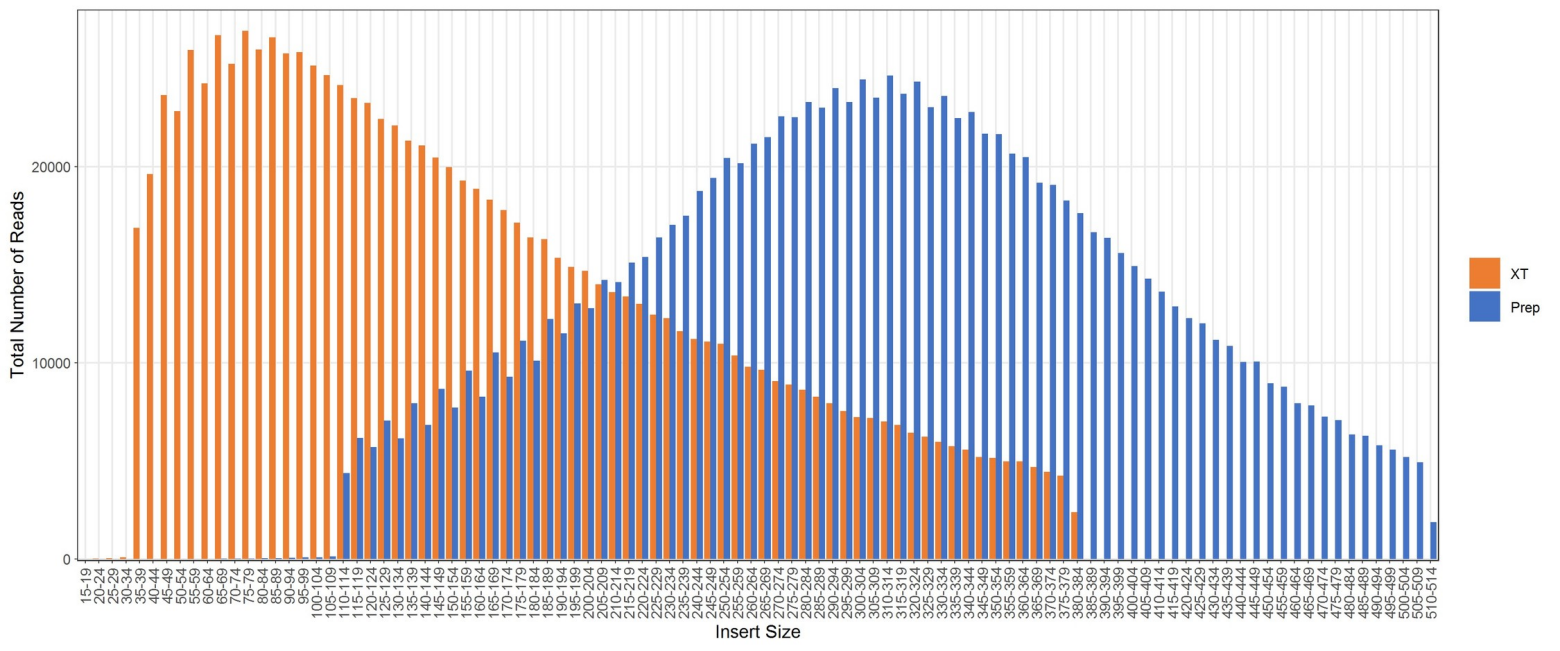
The distribution of the total number of reads based on the size of the paired read length (insert size) for strain CFSAN051560. DNA Prep results are shown in blue and Nextera XT results are shown in orange.

The average read lengths at or above Q30 from DNA Prep were 234 bp out of 250 bp, while the reads from XT were 182 bp out of 250 bp. The average DNA insert size with DNA Prep was 334 bp (ranging from 304 to 370bp) compared to libraries from XT with an average insert size of 260 bp (242 to 354 bp) resulting in less overlap between read 1 and read 2 [4]. Fig 2 shows the distribution of paired read length (insert sizes) for the two library preparations of strain CFSAN051560. The distribution of paired read length (insert sizes) of DNA Prep libraries was consistent across all of the libraries similar to CFSAN051560. In contrast, those from XT showed a higher concentration of smaller insert sizes.

### *In silico* MLST, molecular serotyping and AMRFinder results

Both library preparation kits provided matching *in silico* MLST and molecular serotyping results: all strains belonged to ST 655 and O121:H19, respectively. Assemblies from both library preparations for each sample were interrogated using AMRFinder plus [25] to identify the presence of *E*. *coli* virulence, AMR, and stress tolerance genes. The GenBank protein and nucleotide accession numbers for the genes used by AMRFinder plus can be found at https://www.ncbi.nlm.nih.gov/pathogens/refgene/#. All strains from this outbreak contained the following AMR genes: *acrF*, *blaEC*, and *mdtM*. The strains also contained the following stress genes: *terD*, *terW*, *terZ*, and *ymgB*. These genes were all identified equally by both library preparations. The results are shown in Table 3.

**Table 3:** AMRFinder Plus Results for the 31 STEC strains used in this study by DNA library preparation kit.

Strains	DNA library prep	*efa1*	*espF*	*espI*	*nleC*	*tccP*	*ehxA*	*espP*	*toxB*	PCR-Plasmid^a^
FNW19M81 Reference		**Y**	**Y**	**Y**	**Y**	**Y**	**Y**	**Y**	**Y**	+
CFSAN051458	XT	**Y**	**Y**	**Y**	*N*	**Y**	**Y**	**Y**	*N*	+
	Prep	**Y**	**Y**	**Y**	P	**Y**	**Y**	**Y**	**Y**	+
CFSAN051560	XT	**Y**	**Y**	**Y**	P	P	**Y**	**Y**	*N*	+
	Prep	**Y**	**Y**	**Y**	P	**Y**	**Y**	**Y**	**Y**	+
CFSAN051563	XT	P	**Y**	**Y**	P	**Y**	**Y**	**Y**	*N*	+
	Prep	**Y**	**Y**	**Y**	P	**Y**	**Y**	**Y**	**Y**	+
CFSAN051566	XT	**Y**	**Y**	**Y**	P	P	**Y**	**Y**	**Y**	+
	Prep	**Y**	**Y**	**Y**	P	**Y**	**Y**	**Y**	**Y**	+
CFSAN051758	XT	**Y**	**Y**	**Y**	P	**Y**	**Y**	**Y**	*N*	+
	Prep	**Y**	**Y**	**Y**	P	P	**Y**	**Y**	**Y**	+
CFSAN051761	XT	**Y**	**Y**	**Y**	P	P	**Y**	**Y**	*N*	+
	Prep	**Y**	**Y**	P	P	P	**Y**	P	**Y**	+
CFSAN051762	XT	**Y**	**Y**	**Y**	P	P	**Y**	**Y**	*N*	+
	Prep	**Y**	**Y**	**Y**	P	P	**Y**	**Y**	**Y**	+
CFSAN051763	XT	**Y**	P	**Y**	P	P	P	**Y**	*N*	+
	Prep	**Y**	**Y**	**Y**	P	**Y**	**Y**	**Y**	**Y**	+
CFSAN051765	XT	**Y**	**Y**	**Y**	P	**Y**	**Y**	**Y**	P	+
	Prep	**Y**	**Y**	**Y**	P	**Y**	**Y**	**Y**	**Y**	+
CFSAN051766	XT	**Y**	**Y**	**Y**	P	**Y**	**Y**	**Y**	**Y**	+
	Prep	**Y**	**Y**	**Y**	P	**Y**	**Y**	**Y**	**Y**	+
CFSAN051769	XT	**Y**	**Y**	**Y**	P	**Y**	**Y**	**Y**	P	+
	Prep	**Y**	**Y**	**Y**	P	P	**Y**	**Y**	**Y**	+
CFSAN051772	XT	**Y**	**Y**	**Y**	P	P	**Y**	**Y**	P	+
	Prep	**Y**	**Y**	**Y**	P	**Y**	**Y**	**Y**	**Y**	+
CFSAN052204	XT	**Y**	**Y**	**Y**	P	P	**Y**	**Y**	P	+
	Prep	**Y**	**Y**	**Y**	P	**Y**	**Y**	**Y**	**Y**	+
CFSAN052205	XT	**Y**	**Y**	**Y**	P	P	**Y**	**Y**	P	+
	Prep	**Y**	**Y**	**Y**	P	P	**Y**	**Y**	**Y**	+
CFSAN052206	XT	**Y**	**Y**	**Y**	P	P	**Y**	**Y**	P	+
	Prep	**Y**	**Y**	**Y**	P	**Y**	**Y**	**Y**	**Y**	+
CFSAN052207	XT	**Y**	**Y**	**Y**	P	P	**Y**	**Y**	P	+
	Prep	**Y**	**Y**	**Y**	P	P	**Y**	**Y**	**Y**	+
CFSAN051559	XT	**Y**	**Y**	**Y**	P	**Y**	*N*	*N*	*N*	-
	Prep	**Y**	**Y**	**Y**	P	P	*N*	*N*	*N*	-
CFSAN051561	XT	**Y**	**Y**	**Y**	P	P	*N*	*N*	*N*	-
	Prep	**Y**	**Y**	**Y**	P	P	*N*	*N*	*N*	-
CFSAN051562	XT	**Y**	**Y**	**Y**	**Y**	P	*N*	*N*	*N*	-
	Prep	**Y**	**Y**	**Y**	**Y**	**Y**	*N*	*N*	*N*	-
CFSAN051564	XT	**Y**	**Y**	**Y**	P	P	*N*	*N*	*N*	-
	Prep	**Y**	**Y**	**Y**	P	P	*N*	*N*	*N*	-
CFSAN051565	XT	**Y**	**Y**	**Y**	P	P	*N*	*N*	*N*	-
	Prep	**Y**	**Y**	**Y**	P	**Y**	*N*	*N*	*N*	-
CFSAN051567	XT	**Y**	**Y**	**Y**	P	P	*N*	*N*	*N*	-
	Prep	**Y**	**Y**	**Y**	P	P	*N*	*N*	*N*	-
CFSAN051568	XT	**Y**	**Y**	**Y**	P	**Y**	*N*	*N*	*N*	-
	Prep	**Y**	**Y**	**Y**	P	**Y**	*N*	*N*	*N*	-
CFSAN051759	XT	**Y**	**Y**	**Y**	P	P	*N*	*N*	*N*	-
	Prep	**Y**	**Y**	**Y**	P	**Y**	*N*	*N*	*N*	-
CFSAN051760	XT	**Y**	**Y**	**Y**	P	**Y**	*N*	*N*	*N*	-
	Prep	**Y**	**Y**	**Y**	P	P	*N*	*N*	*N*	-
CFSAN051764	XT	**Y**	**Y**	**Y**	P	P	*N*	*N*	*N*	-
	Prep	**Y**	**Y**	**Y**	P	P	*N*	*N*	*N*	-
CFSAN051767	XT	**Y**	**Y**	**Y**	**Y**	P	*N*	*N*	*N*	-
	Prep	**Y**	**Y**	**Y**	P	**Y**	*N*	*N*	*N*	-
CFSAN051768	XT	**Y**	**Y**	**Y**	P	P	*N*	*N*	*N*	-
	Prep	**Y**	**Y**	**Y**	P	P	*N*	*N*	*N*	-
CFSAN051770	XT	**Y**	**Y**	**Y**	P	P	*N*	*N*	*N*	-
	Prep	**Y**	**Y**	**Y**	P	**Y**	*N*	*N*	*N*	-
CFSAN051771	XT	**Y**	**Y**	**Y**	P	P	*N*	*N*	*N*	-
	Prep	**Y**	**Y**	**Y**	P	**Y**	*N*	*N*	*N*	-
CFSAN052208	XT	**Y**	**Y**	**Y**	P	**Y**	*N*	*N*	*N*	-
	Prep	**Y**	**Y**	**Y**	P	**Y**	*N*	*N*	*N*	-

Genes in gray are found on the plasmid. Y = Identified, N = Not identified, P = Partial identification. All strains were positive for *acrF*, *blaEC*, *mdtM*, *terD*, *terW*, *terZ*, *ymgB eae*, *stx2a*, *espA*, *espB*, *espJ*, *espK*, *fdeC*, *lpfA*, *nleA*, *nleB*, and *tir*.

^a^PCR specific targeting the *ehxA* gene in the plasmid. If present + and not present -.

In the case of the virulence genes, AMRFinder Plus identified that these strains carried intimin subtype epsilon and *stx2a* in all assemblies (Table 3). For those virulence genes found on the chromosome, there were two genes (*nleC* and *tccP*) that could only be partially identified a majority of the time, regardless of library preparation. Although these genes were a “partial identification”, meaning more than 50% of the sequence is identified, we regarded them as being positive for the presence of the gene. The main difference in virulence gene detection was in the genes found on the plasmid (*ehxA*, *espP*, *toxB*).

Interestingly, a subset of 18 strains did not appear to have any of the three virulence genes expected to be found in the plasmid. As Table 3 illustrates, neither library preparation method was able to detect these genes, and we considered it possible that these 18 strains lacked the virulence plasmid entirely. Two possible explanations could be that these strains either may had lost the plasmid during serial culture in our laboratory or two populations (one with the plasmid and one without) had always been present among our isolates. To explore this hypothesis, a previously reported PCR screen for detecting the *ehxA* gene was utilized [14]. *In silico* analyses of the strains that identified *ehxA*, as well as the closed strain FNW19M81, were used as the positive controls in the PCR. The *ehxA* positive strains resulted in a PCR product of the correct size (~1500 bp) while the *ehxA* negative strains by *in silico* WGS analyses resulted in no PCR product. We believe the failure to detect the *ehxA* PCR products in the 18 strains, along with the *in silico* results of the other two plasmid virulence genes (*espP* and *toxB*), confirm these strains do not carry the plasmid.

**Fig 3:**
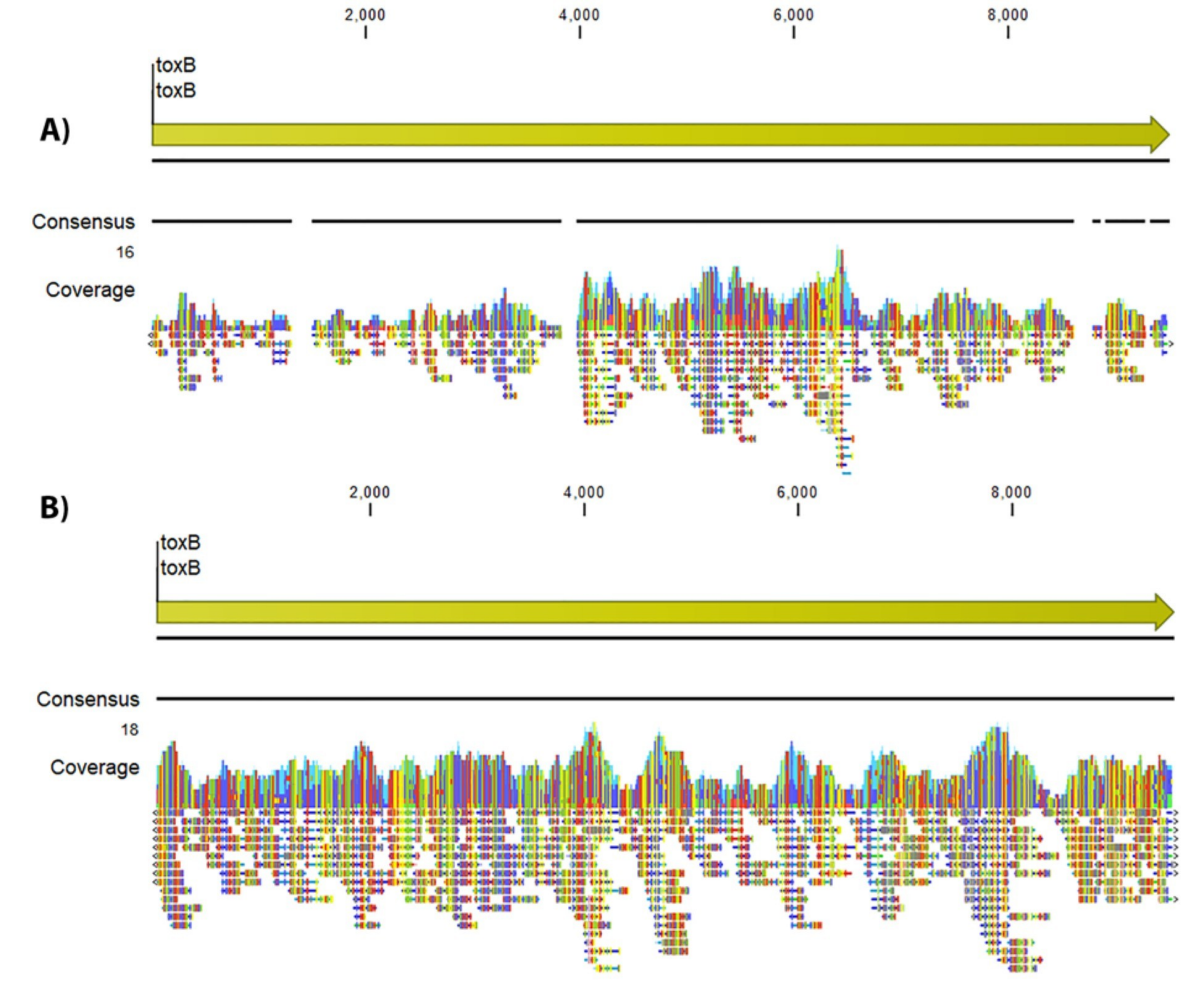
Reference mapping of the *toxB* gene (we used the *toxB* gene sequence from strain Sakai O157:H7 as reference). A) Nextera XT output for strain FNW19M81 showing that the reads did not cover the entire gene and therefore that gene was not present in the final XT assembly. B) Nextera DNA Prep output for the same strain showing that it covered the entire *toxB* gene and therefore that gene was present in the final Prep assembly.

The XT libraries identified *ehxA* in all 15 strains that carried the plasmid (93.3% complete and 6.7% partial), *espP* in all strains (100% complete), and *toxB* in nine strains (13.3% complete and 46.7% partial). The libraries created using DNA Prep had higher rates of identification of the plasmid genes: *ehxA* in all strains (100% complete), *espP* in all strains (93.3% complete and 6.7% partial), and *toxB* in all strains (100% complete). As shown in Fig 3, the raw reads and therefore the assemblies from XT libraries did not cover the entire *toxB* gene, unlike the raw reads from DNA Prep by using read mapping and visualization in CLC Genomics Workbench.

### SNP phylogenetic analysis

**Fig 4:**
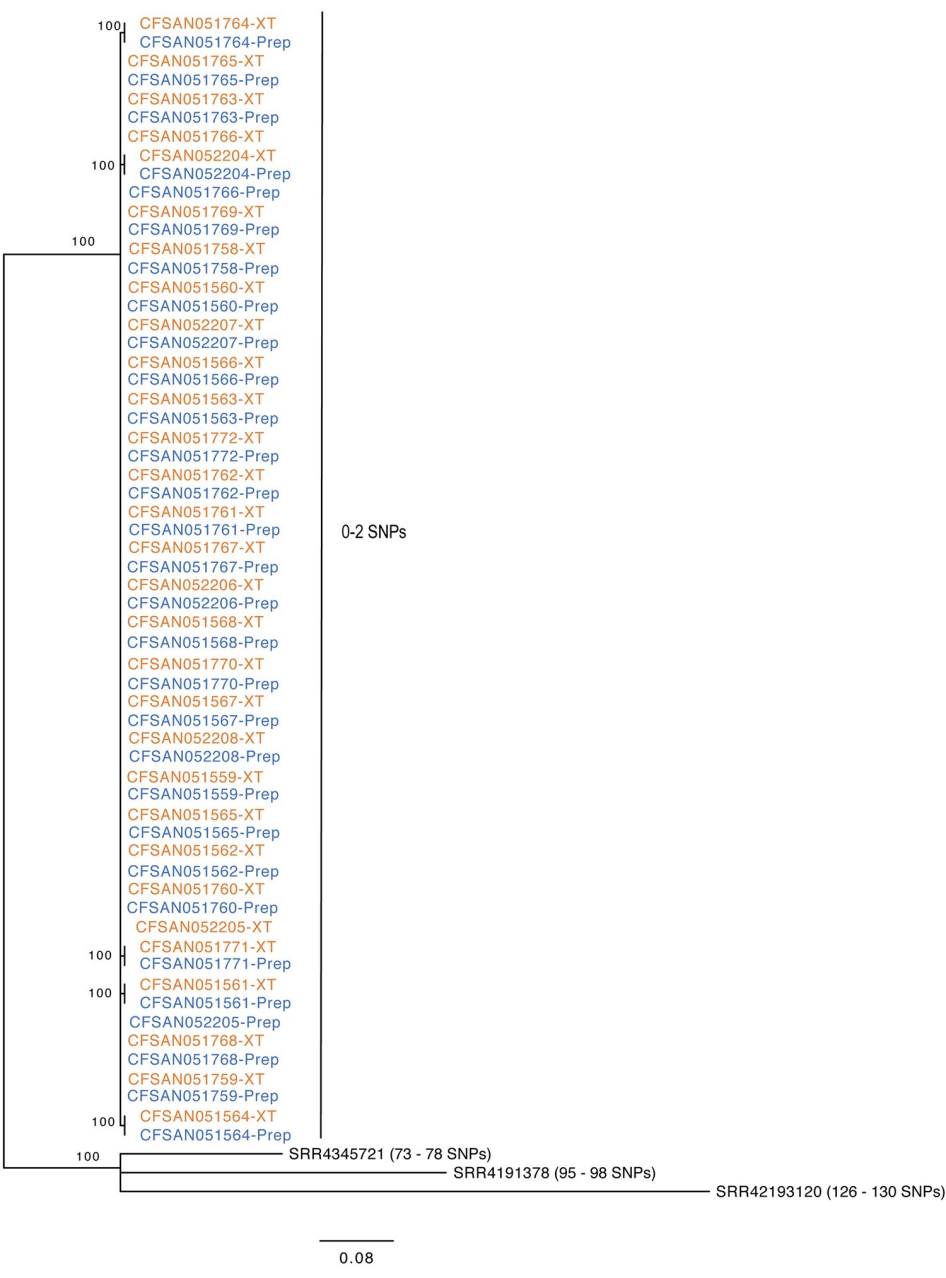
Phylogenetic tree obtained by a maximum-Likelihood analysis of the resultant SNP matrix from the CFSAN SNP pipeline of the data from the 30 strains obtained from each DNA library kit. Results obtained with the XT libraries are shown in red while DNA Prep libraries are show in blue. The genome of FNW19M81 was used as the reference.

To test that both library preparations produced the same phylogenetic result (SNP calls), the CFSAN SNP pipeline [27] was used to analyze the raw data for all strains from both library preparations using FNW19M81 as the reference and three near-neighbor clinical isolates that were unrelated to the outbreak as outliers. The maximum likelihood tree generated by the SNP matrix is shown in Fig 4. All 30 of the flour isolates clustered together and were separated by 0–2 SNPs. The three unrelated clinical isolates were separated by 73–130 SNPs from the flour cluster. There were no SNP differences identified between the different library preparation methods.

## Discussion

Whole genome sequencing is comprised of many different modules; how each of those modules perform may influence the end results [29, 30]. Any artifacts arising during bacterial culture and DNA extraction can potentially distort the resultant library and sequence data, and on through all subsequence analyses. To reduce such interference, our study used the same DNA extracts from the *E*. *coli* strains, collected during the 2016 outbreak, to allow direct comparisons of the data results obtained from the XT and DNA Prep chemistries. We found the DNA Prep kit generated higher quality data and better genome coverage, corroborating the findings of previous studies using the DNA Prep method: the bead-linked transposome improves coverage uniformity in regions that are often difficult to sequence [2, 4]. Bruinsma, et. al (2019), described the findings that the DNA Prep kit resulted in more uniform insert sizes and concentration of the final libraries. They also stated the DNA Prep kit improved the sequencing of organisms with variable GC content and allowed an even distribution of read depth across the genome [4]. Furthermore, our study shows similar findings regarding a more consistent coverage of the complete genome of the isolates. The importance of complete WGS of STEC cannot be understated for public health importance, making the library preparation method extremely crucial to ensure complete and accurate sequencing.

One advantage of the DNA Prep kit is that the bead-linked enzyme controls the tagmentation process thus the median insert size in the resulting library. Not only were the average insert size distributions larger for DNA Prep, there is overall more stability in the quality of the libraries prepared using this kit compared to XT (335 ± 17.8 bp and 260 ± 28.8 bp, respectively). The larger, more stable insert size using DNA Prep allowed for less sequencing overlap (more bases of the genome to be sequenced), leading to increased genome breadth coverage, and overall better assemblies. Overall, the depth of sequencing does not seem to have as much impact on the genome size whereas the quality of sequencing data does. For DNA prep libraries of isolates that contain the plasmid the total genome size is up to 91,000 base pairs larger than XT libraries. The difference in genome size is reduced in isolates that are lacking the plasmid with the largest difference being 36,000 base pairs.

The MiSeq output for DNA Prep runs (n = 2) showed a Q30 for the run above 90% while the XT runs (n = 3) had a Q30 below 80%. Overall, the MiSeq run quality for libraries prepared with DNA Prep had higher Pass Filter % of reads when compared with the runs of libraries prepared with XT. This impact can be seen in the average read length at or above Q30; higher Q30 average length leads to better quality assemblies and this metric reflects the difference in overall quality of Prep vs XT (Fig 1). These factors combined shows the robust and consistent nature of the DNA Prep library preparation. Additionally, the amount of time needed to prepare a library is comparable between the two kits, even with addition of a dual size selection step (for the DNA Prep kit). The Prep libraries are self-normalized when using high DNA inputs (>100 ng) and the concentration of input DNA is less critical compared to the XT kit, which saves time during the initial quantification and dilutions. The DNA Prep kit also allows researchers to customize the target insert size. The Prep libraries provide larger insert size, higher Q30, and better quality reads, all of which contribute to having fewer run failures and more accurate assemblies (fewer contigs with higher N50 length and larger genome sizes).

Rapid and accurate detection of virulence genes is an essential part of characterizing any *E*. *coli* connected to outbreaks of illness. In some outbreaks, such as the *E*. *coli* O104:H4 outbreak in 2011, which centered in Germany, but also affected people in other European Union nations [31, 32], the outbreak strain exhibited a unique combination of virulence genes. The virulence genes present resulted in a strain that had unusual pathogenicity and outcomes in human illnesses, which led to a much higher proportion of affected people developing hemolytic uremia syndrome (HUS) (~23% HUS cases, compared to 6% among classic rates of HUS for STECs) [33]. This O104:H4 strain belonged to ST678, and produced Stx2a, but WGS revealed that this strain was 93% identical to enteroaggregative *E*. *coli* (EAEC) strain 55589 [33–35]. Taken together the genetic analyses revealed that this strain was an EAEC strain that had acquired the ability to produce Stx via phage conversion. Identification of such plasmid-borne genes is a necessity, not only during major outbreaks, but also for routine surveillance of AMR plasmids, which could be acquired by bacteria via uptake or horizontal transfer. The DNA Prep library kit allows these plasmids to be sequenced, thereby increasing our ability to perform critical public health surveillance.

The method of library preparation impacts the ability to identify all virulence genes, including those which present unique genomic challenges. The aforementioned 2011 outbreak emphasizes the necessity for a library preparation method which is able to capture the entire genome in the data. In this study, one of the largest discrepancies between library preparations was seen regarding the *toxB* gene. The *toxB* is a 10-kb virulence gene that is distributed among enterohemorrhagic *E*. *coli* (EHEC) and enteropathogenic *E*. *coli* (EPEC) [36]. While the Prep assemblies were able to identify this gene 100% of the time, the assemblies from XT only identified this gene 58% of the time. Several factors may contribute to this discrepancy: identification of the gene may be affected by its size, low GC content (31%), and its position–it is surrounded by insertion sequences–each of these factors can make identification more difficult. Although plasmids can be difficult to capture with WGS, we believe the increased amount of DNA input, coupled with the lack of bias during the fragmentation process, allows more of the plasmid to be sequenced.

The phylogenetic tree generated from the SNP analysis shows that phylogenetic analysis can be performed on libraries from either kit and provide similar results. This allows for phylogenetic analysis irrespective of library preparation method. Retrospective data of libraries from XT preparations can be analyzed along with Prep libraries which provides for concordance with historical data.

## Conclusion

Our preliminary results suggest that there are benefits to using DNA Prep library preparation for virulence typing in *E*. *coli*. The primary decision of which library kit to use becomes more important for the detection of virulence genes and what the goal of the analyses are. Due to the higher and more consistent DNA input, the plasmid coverage is enhanced, and the overall coverage of the chromosome is evenly distributed. Virulence genes were identified at a higher rate and overall run quality was improved by utilizing the DNA Prep library preparation method. In contrast to the XT preparation, results from the DNA Prep kit showed more consistent insert size regardless of GC content or DNA input.
